# Supplementary material for: Caregiver Knowledge, Attitude, and Behavior toward Care of Children with Cerebral Palsy: A Saudi Arabian Perspective
Source: Healthcare (Basel). 2024 May 10;12(10):982. doi: 10.3390/healthcare12100982 (PMC11121316; doi:10.3390/healthcare12100982)
Supplement: Supplementary file 1 [file healthcare-12-00982-s001.zip › healthcare-2953477-supplementary.pdf]

Supplementary material

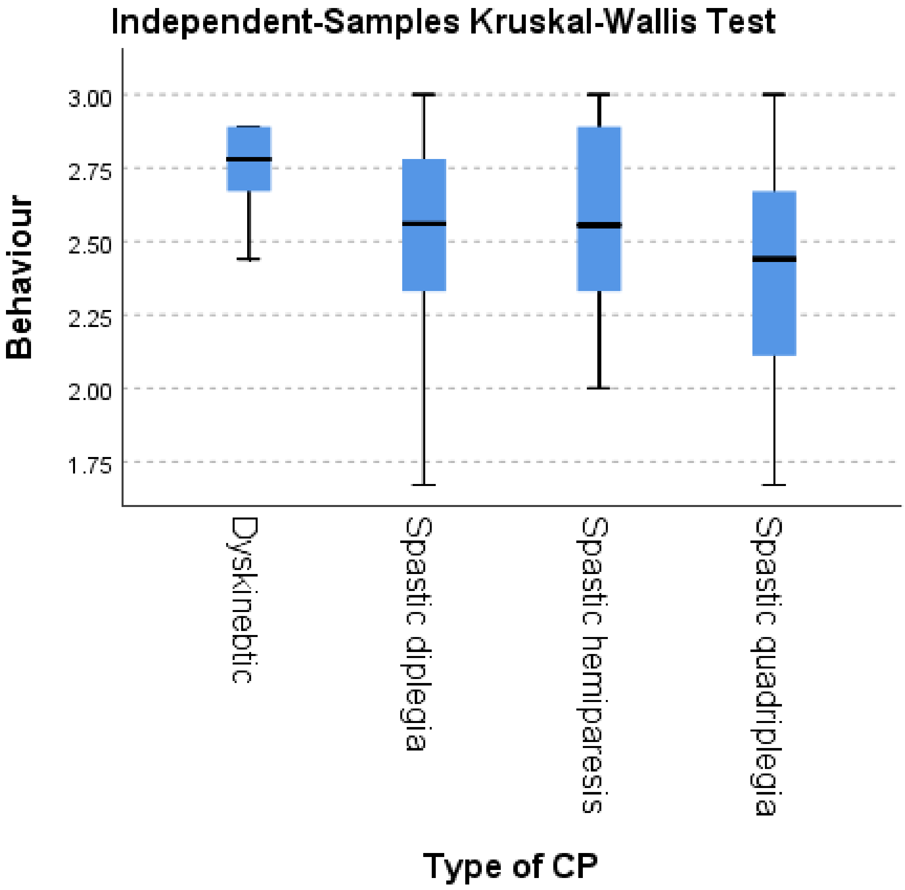

Figure S1. Type of CP.

Table S1. Pairwise Comparisons of Type of CP.

Sample 1-Sample 2	Test Statistic	Std. Error	Std. Test Statistic	Sig.	Adj. Sig.[a]
Spastic quadriplegia-Spastic diplegia	19.602	8.863	2.212	0.027	0.162
Spastic quadriplegia-Spastic hemiparesis	27.329	17.747	1.540	0.124	0.741
Spastic quadriplegia-Dyskinebtic	55.765	28.499	1.957	0.050	0.302
Spastic diplegia-Spastic hemiparesis	-7.727	17.780	-0.435	0.664	1.000
Spastic diplegia-Dyskinebtic	36.163	28.520	1.268	0.205	1.000
Spastic hemiparesis-Dyskinebtic	28.436	32.401	0.878	0.380	1.000

Each row tests the null hypothesis that the Sample 1 and Sample 2 distributions are the same.
A symptotic significances (2-sided tests) are displayed. The significance level is .05.
a. Significance values have been adjusted by the Bonferroni correction for multiple tests.

Table S2. Behavior related to age of the CP child and type of CP

**Model**		**Unstandardized Coefficients**		Standardized Coefficients	t	Sig.	95.0% Confidence Interval for B	
		**B**	**Std. Error**	Beta			Lower Bound	Upper Bound
1	(Constant)	2.814	0.102		27.501	0.000	2.612	3.016
	Age of CP child	-0.102	0.048	-0.141	-2.115	0.036	-0.197	-0.007
	Type of CP	-0.059	0.024	-0.163	-2.443	0.015	-0.106	-0.011

Coefficients[a]

a. Dependent Variable: Behaviour
